# Association Between HbA1c Levels on Adverse Pregnancy Outcomes During Pregnancy in Patients With Type 1 Diabetes

**DOI:** 10.1210/clinem/dgab769

**Published:** 2021-10-25

**Authors:** Madleen Lemaitre, Camille Ternynck, Julien Bourry, Florence Baudoux, Damien Subtil, Anne Vambergue

**Affiliations:** 1 Department of Diabetology, Endocrinology, Metabolism, and Nutrition, CHU Lille, Lille University Hospital, Lille, France; 2 Department of Medicine, University of Lille, France; 3 , ULR 2694-METRICS: évaluation des technologies de santé et des pratiques médicales, University of Lille, CHU Lille, Lille, France; 4 Department of Biostatistics, CHU Lille, Lille, France; 5 Department of Gynecology and Obstetrics, CHU Lille, Lille University Hospital, Lille, France; 6 European Genomic Institute for Diabetes, University School of Medicine, Lille, France

**Keywords:** type 1 diabetes, pregnancy, HbA1c, adverse pregnancy outcomes

## Abstract

**Context:**

Despite optimization of metabolic balance during pregnancy in type 1 diabetes (T1D), maternal–fetal complications remain higher than in the background population.

**Objective:**

We examined whether there is an association between glycated hemoglobin (HbA1c) levels and these complications.

**Methods:**

Retrospective study of pregnancies in 678 T1D subjects at Lille Hospital (1997-2019). The association between variations in HbA1c levels and complications was examined. The composite criterion (CC) was defined as having at least 1 of the following complications: prematurity, pre-eclampsia, large for gestational age (LGA), small for gestational age (SGA), or cesarean section.

**Results:**

Among the 678 births, median preconception HbA1c was 7.2% (55 mmol/mol), 361 were LGA (56%), 29 were SGA (4.5%), and 504 were births without preterm delivery (76.1%). The CC occurred in 81.8%. Higher HbA1c during the first trimester was associated with the CC (OR 1.04; 95% CI 1.02-1.06 per 0.1% increase; *P* < .001). Higher HbA1c during the third trimester was associated with the CC (OR 1.07; 95% CI 1.03-1.10 per 0.1% increase; *P* < .001). The group defined by a first trimester Hba1c >6.5% (48 mmol/mol) and a third trimester HbA1c <6% was associated with an increased rate of the CC (OR 2.81; 95% CI 1.01-7.86) and an increased rate of LGA (OR 2.20; 95% CI 1.01- 4.78).

**Conclusion:**

Elevated HbA1c is associated with maternal–fetal complications. Despite optimization of metabolic balance during the third trimester, for patients with early glycemic imbalance the risk of LGA persists.

The prevalence of diabetes during pregnancy, mostly gestational diabetes, continues to rise worldwide (1). However, maternal–fetal risks, in terms of morbidity and mortality, are predominantly present in diabetes that is pre-existent at the time of pregnancy, with an even higher risk of complications in type 1 diabetes (T1D) (2). The most common complications are congenital malformations, stillbirth or neonatal death, macrosomia, in utero growth retardation, shoulder dystocia, neonatal hypoglycemia, and neonatal respiratory distress, which may lead to admission to a neonatal intensive care unit (3).

In 1989, the goal that pregnancy outcomes in women with pre-existing diabetes at the time of pregnancy should be similar to those of the background population was established (4). Despite increasingly strict management, this objective remains elusive. In 2015, an observational cohort study described a higher risk of pre-eclampsia, cesarean section, stillbirth, congenital malformations, and prematurity in women with diabetes (5). More recently, the CONCEPTT study reported that maternal–fetal complications can be improved by better glycemic control, but even with the improvement obtained with continuous glucose monitoring (CGM) pregnancy outcomes remained suboptimal, with a high proportion of infants with macrosomia and high levels of neonatal morbidity (6).

Pregnancy should be anticipated and planned in such cases to limit the risk of adverse outcomes. Indeed, the American Diabetes Association recommends a preconception glycated hemoglobin (HbA1)c level between 6% (42 mmol/mol) and 6.5% (48 mmol/mol), while limiting, as much as possible, the number of hypoglycemic episodes, and advocates preconception counseling and programming of pregnancy, the benefits of which have already been shown (7). During pregnancy, HbA1c levels have been shown to be a strong predictor of maternal and fetal complications (8). The ideal HbA1c level during pregnancy is likely to be lower than the above values since HbA1c decreases during the first and second trimesters, linked to pathophysiological changes (9). O’Connor et al. defined a normal range for HbA1c in pregnant Caucasian women as <5.4% (36 mmol/mol) in the first trimester, <5.4% (36 mmol/mol) in the second trimester, and <5.7% (39 mmol/mol) in the third trimester (10).

The aim of our study was to examine whether there is an association between HbA1c levels and maternal–fetal complications in T1D who were followed in the same tertiary obstetric care center by the same multidisciplinary team.

## Materials and Methods

### Research Design

This single-center observational study was performed at the University hospital of Lille, France based on electronic records, including the metabolic and obstetric data that are routinely collected at delivery for every birth. Under French law, care-related data may be used for research purposes unless the patient opposes such use. Data were analyzed anonymously, and our database was declared to the French Committee for computerized data. In this observational cohort, we included all women with pregestational diabetes who gave birth between 1997 and 2019. All pregnancies were analyzed, but only pregnancies of women with T1D were included. Patients were excluded if they were under 18 years old or had other types of diabetes, including type 2 diabetes, monogenic diabetes, syndromic diabetes, or secondary diabetes. Additional exclusion criteria included lack of data or consent, persistent doubt regarding diagnosis, lost to follow-up, or twin/multiple pregnancies since these had a higher risk of adverse outcomes.

### Study Population and Outcomes Definitions

Maternal demographics, obstetric and ophthalmologic examinations, time of diabetes diagnosis, and the presence of complications were collected from patient charts. Diabetes and obstetric follow-up were performed monthly and patients contacted twice a week by a specialized nurse to assess glycemic control and adjust the insulin dose where needed. Patients were treated with short-acting insulin analogs before meals and long-acting insulin analogs in the morning and/or at bedtime, or with continuous subcutaneous insulin infusion (CSII). We have followed the French guidelines which recommended self–blood glucose monitoring with a glucose target <100 mg/dL before meals and <140 mg/dL after meals (11).

Age, height, and body weight were recorded and body mass index (BMI) was calculated in kg/m^2^. Blood pressure was measured, with hypertension defined as >140/90 mmHg or the use of an antihypertensive drug before pregnancy. Diabetes history was recorded including duration of diabetes, therapy used (multiple daily injections [MDIs] or CSII), preconception HbA1c, and vascular complications: history of nephropathy (albuminuria ≥30 mg/24 hours or renal insufficiency), history and status of retinopathy.

Obstetric history was assessed: parity, gravidity, date of pregnancy, history of macrosomia, hypertension, pre-eclampsia, miscarriage, or stillbirth. Administration of a daily dose of 5 mg of folic acid was started as soon as conception was planned, and continued until week 12 weeks’ gestation. Losses of pregnancy were recorded: miscarriage was defined as the loss of pregnancy before 24 weeks’ gestation. Stillbirth was defined as fetal loss occurring after 24 weeks’ gestation. Hypertension was defined as the appearance or aggravation of hypertension. Pre-eclampsia was defined as association of systolic blood pressure >140 mmHg or diastolic blood pressure >90 mmHg and proteinuria ≥300 mg/24 hours after 20 weeks of amenorrhea.

Prematurity was defined as birth prior to 37 weeks of amenorrhea. Delivery modality was recorded (vaginal or cesarean section). Induction of labor was systematically performed between 38 and 39 weeks’ pregnancy, in accordance with the recommendations of the CGNOF (French Society of Obstetrics).

Birthweight (BW) was used to define macrosomia as BW ≥4000 g. BW was adjusted for neonatal sex and gestational age for singleton pregnancies using customized percentiles, with large for gestational age (LGA) defined as birthweight centile above the ninetieth percentile and small for gestational age (SGA) defined as below the tenth percentile (AUDIPOG curves) (12).

A composite criterion (CC), which associated preterm delivery, pre-eclampsia, LGA, SGA, and cesarean section, was defined to estimate the proportion of maternal–fetal morbidity in our population. This criterion was considered positive if at least 1 component was present.

HbA1c was measured monthly using automated high-pressure liquid chromatography in the period 1997-2015. After 2015, capillary electrophoresis was performed (Capillaris Tera SEBIA, normal range 4.0-6.0% [20-42 mmol/mol]; coefficient of variation <3%). Assay performance was certified by Bio Rad. HbA1c was measured during the first month then during the first (<15 weeks’ gestation), second (< 28 weeks’ gestation), and third trimesters (<41 weeks’ gestation). For the statistical analysis, we performed a mean of each HbA1c taken every trimester. Delta HbA1c first – third trimester was defined as the difference in the means of HbA1c in the first trimester compared with means in the third trimester. We compared the CC and most frequent individual adverse outcomes (LGA, prematurity, cesarean section) among 4 glycemic control subgroups defined by HbA1c levels in the first and third trimesters: group I, <6.5% in the first trimester and <6.0% in the third trimester; group II, ≥ 6.5% in the first trimester and <6.0% in the third trimester; group III, <6.5% in the first trimester and >6.0% in the third trimester; group IV, ≥6.5% in the first trimester and ≥6.0% in the third trimester.

### Statistical Analysis

Statistical analyses were conducted using SAS software (SAS Institute 9.4, Cary, USA). Categorical variables were reported as numbers (percentage). Quantitative variables were described as mean ± SD, in the case of Gaussian distribution, or otherwise by median (interquartile range [IQR]). Normality of numerical variables was checked graphically and tested using the Kolmogorov–Smirnov test. We assessed the association of HbA1c (assessed at the first and third trimesters, as well as the difference between first and third trimester values) with pregnancy outcomes (CC and individual adverse events) using logistic regression models before and after adjustment for year of the date of pregnancy, or for treatment. Comparisons were made using logistical regression models before and after adjustment on period of date of pregnancy, using subgroup I as reference. All results were expressed as OR and their 95% CI. For CC outcome, the predictive ability of HbA1c, assessed in the first and third trimesters, was evaluated by receiver operating characteristic (ROC) curve analysis, by calculating the area under the ROC curve (AUC) and its 95% CI. Statistical testing was 2-tailed with *P* < .05 accepted as statistically significant.

## Results

### Demographic Characteristics of Type 1 Diabetic Population

**Figure 1.**
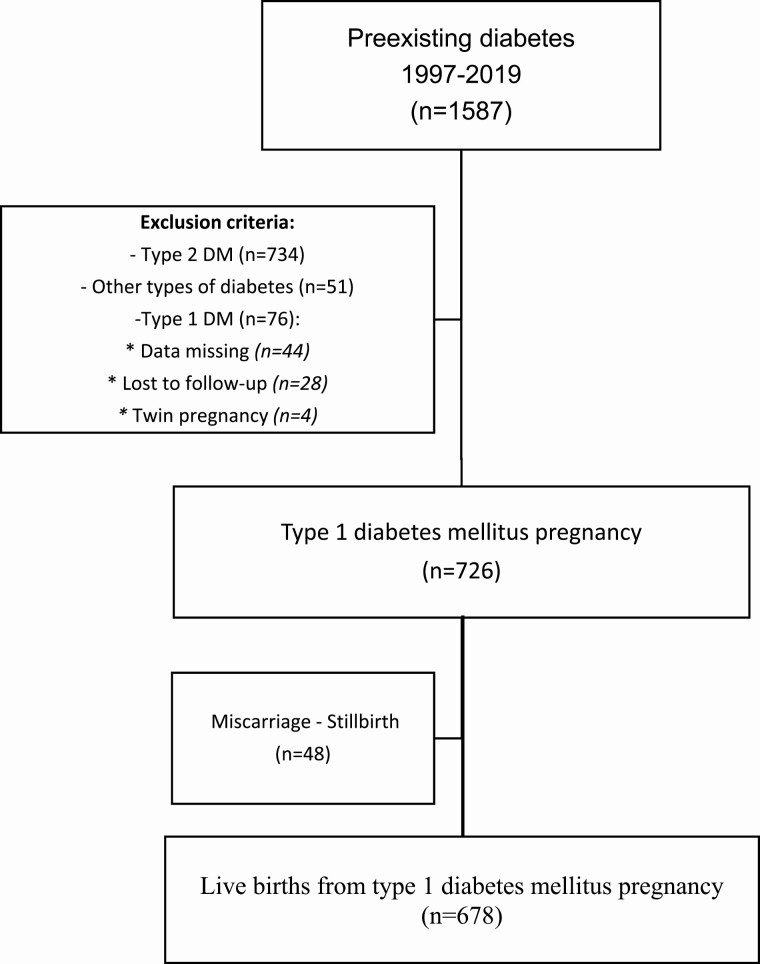
Patient enrollment flowchart. DM, diabetes mellitus; FDIU, fetal death in utero.

During the study period, we included 1587 pregnancies with maternal diabetes. We excluded 861 pregnancies: 734 with type 2 diabetes, 51 with other forms of diabetes, 76 women with T1D because data were missing (n = 44), lost to follow-up (n = 28), and twin pregnancy (n = 4). So in this study, 726 pregnancies, among 510 patients, were included in our study: 348 with 1 pregnancy, 119 with 2 pregnancies, 32 with 3 pregnancies, and 11 with 4 or more pregnancies. Forty-eight pregnancies were excluded due to miscarriage or stillbirth so the data were analyzed in 678 live birth (Fig. 1).

### Baseline Maternal Characteristics

The clinical characteristics are presented in Table 1. The mean age was 30.1 (± 4.1) years, median preconception BMI was 23.5 kg/m^2^ (IQR 21.3-26.7). The median duration of diabetes was 14 years (IQR 7-20). Before pregnancy, 373 (55%) of women were treated using CSII, with only 163 (25.3%) having HbA1c ≤6.5%. Only 194 (28.7%) had diabetic vascular complications: diabetic nephropathy was present in 44 women (6.5%), diabetic retinopathy in 177 (26.1%), and pregravid hypertension in 25 (3.7%).

**Table 1.** Baseline maternal characteristics

	Total (n = 678)
Age, years	30.1 ± 4.8
Body mass index^*a*^, kg/m^2^	23.5 (21.3-26.7)
Duration of diabetes^*b*^, years	14 (7-20)
Multiple daily injections	305/678 (45)
Continuous subcutaneous insulin infusion	373/678 (55)
Prepregnancy glycated hemoglobin, %	7.2 (6.5-8.1)
No complication	462/632 (73.1)
Diabetic nephropathy	44/677 (6.5)
Hypertension	25/676 (3.7)
Nulliparity	260/677 (38.4)
History of macrosomia	85/672 (12.6)

Values are expressed as the number/total number (%), mean ± SD or median (interquartile range).

^*a*^45 missing values.

^*b*^5 missing values.

### Adverse Maternal–Fetal Outcomes During Pregnancies in T1D

Maternal and fetal adverse outcomes are presented in Table 2. Concerning maternal complications, 77 (11.5%) presented gravid hypertension and 48 (7.2%) presented pre-eclampsia. In terms of vascular diabetic complications, 165 women (25.1%) had an appearance or aggravation of diabetic retinopathy including 127 de novo cases (77% of them), and 124 (18.6%) had appearance or aggravation of diabetic nephropathy including 79 de novo cases (64% of them). Among the 726 studied pregnancies with T1D, 48 (6.6%) ended in miscarriage or stillbirth.

**Table 2.** Maternal and fetal adverse pregnancy outcomes

	Total (N = 678)
Gestational age at delivery^*a*^, weeks	38 (37-38.2)
Gestational hypertension	77/667 (11.5)
Pre-eclampsia	48/668 (7.2)
Prematurity	159/663 (24)
Cesarean section	320/666 (48)
Birth weight^*b*^, grams	3530 (3160-3900)
Macrosomia	130/663 (19.6)
LGA	361/645 (56)
SGA	29/647 (4.5)
Shoulder dystocia	64/665(9.6)
Ketoacidosis	16/616 (2.8)
Neonatal malformations	39/663 (5.9)
NICU admission	58/667(8.7)
Composite criterion of complications^*c*^	536/655 (81.8)

Values are expressed as the number/total number (%), mean ± SD or median (interquartile range).

Abbreviations: LGA, large for gestational age; SGA, small for gestational age; NICU, Neonatal Intensive Care Unit.

^*a*^15 missing values.

^*b*^27 missing values.

^*c*^The composite criterion of complications is defined by the presence of at least 1 of the 5 following complications: pre-eclampsia, LGA, SGA, cesarean, prematurity.

Concerning fetal complications, the median gestational age at delivery was 38 weeks (IQR 37-38.2). The prematurity rate was 24% (n = 159). Mean birthweight was 3484 (± 675.6) grams. The rate of macrosomia and LGA was 19.5% and 56%, respectively. The rate of SGA was 4.5%. Shoulder dystocia was described in 9.6% of children and 8.7% of children were admitted to the Neonatal Intensive Care Unit; 5.9% of children showed a neonatal malformation. The CC, defined by at least 1 complication among pre-eclampsia, LGA, SGA, cesarean section and prematurity, was present in 81% of cases.

### HbA1c and Pregnancy Outcomes

**Figure 2.**
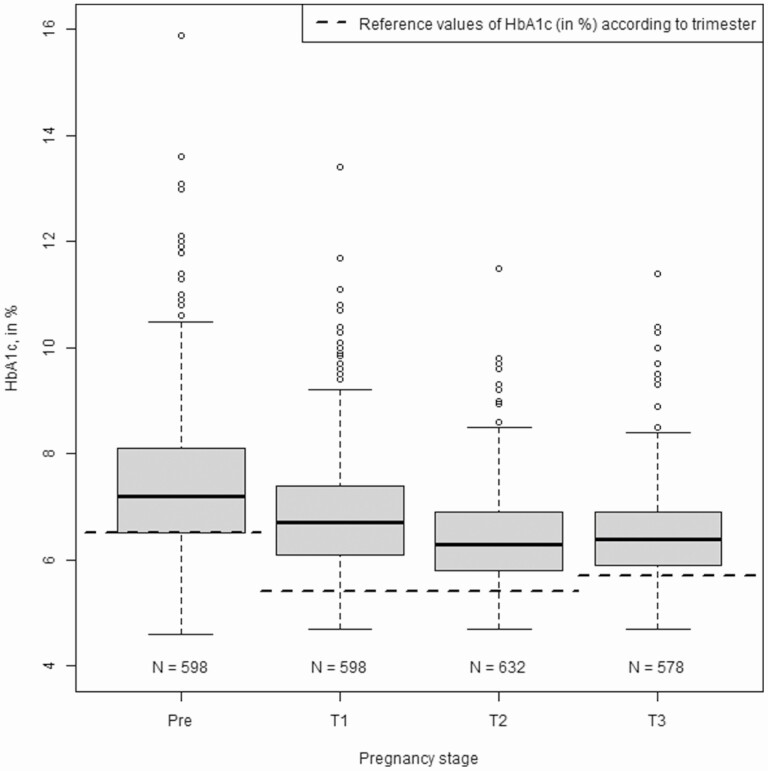
Distribution (Tukey’s boxplots) of HbA1c according to pregnancy stage during type 1 diabetes pregnancies. HbA1c is expressed as percentage. Before pregnancy, recommended HbA1c is <6.5%. During pregnancy, HbA1c is considered as normal for values <5.4% in the first and second trimester and values <5.7% in the third trimester [10]. Reference values of HbA1c (in %) according to trimester is represented by the broken black line (- - -).

The mean HbA1c level during pregnancy was 6.6% (49 mmol/mol) (IQR 6.1-7.2) (Fig. 2). Prepregnancy HbA1c was 7.2% (55 mmol/mol) (IQR 6.5-8.1). As expected, HbA1c values decreased during pregnancy: median HbA1c in the first month was 7% (53 mmol/mol) (IQR 6.4-8.1), 6.7% (50 mmol/mol) (IQR 6.1-7.4) in the first trimester, 6.3% (45 mmol/mol) (IQR 5.8-6.9) in the 2nd trimester, and increased to 6.4% (46 mmol/mol) (IQR 5.9-6.9) in the third trimester.

**Figure 3.**
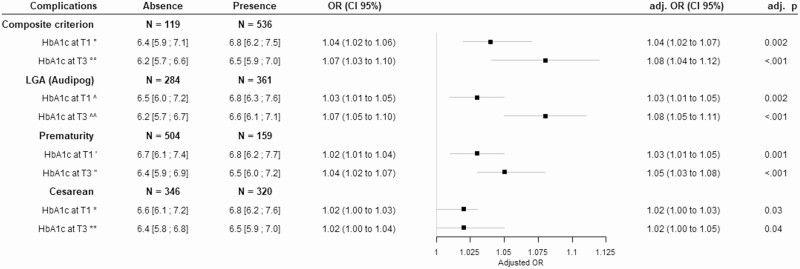
Association of composite criterion and most individual adverse events with HbA1c during type 1 diabetes pregnancy (adjusted for years of the date of pregnancy). Results are expressed as median (interquartile range), or OR (95% CI) without and with adjustment for year of pregnancy and as adjusted *P* value. The composite criterion of complications is defined by the presence of at least one of the five following complications: preeclampsia, LGA, SGA, cesarean, prematurity. °77 missing values (respectively 8 and 69); °°94 missing values (respectively 13 and 81). ^74 missing values (respectively 29 and 45); ^^89 missing values (respectively 39 and 50). ˈ78 missing values (respectively 54 and 24); ˈˈ95 missing values (respectively 54 and 41); *76 missing values (respectively 36 and 40); **93 missing values (respectively 40 and 53). OR, odds ratio; CI, confidence interval; LGA, large for gestational age; SGA, small for gestational age; T1, trimester 1; T3, trimester 3.

Higher HbA1c during the first trimester was associated with the CC (OR 1.04; 95% CI 1.02-1.06 per 0.1% increase; *P* = .001) (Fig. 3). Higher HbA1c during the third trimester was also associated with the CC (OR 1.07; 95% CI 1.03-1.10 per 0.1% increase; *P* < .001). After adjustment for year of the date of pregnancy, these associations remained. After adjustment for treatment, these associations remained (Supplementary data 1 (13)). ROC curve analysis showed the low predictive ability of HbA1c on the CC with an AUC of 0.62 (95% CI 0.56-0.67) in the first trimester and an AUC of 0.63 (95% CI 0.58-0.69) in the third trimester. The cut-off values obtained were 6.46% for the first trimester (sensitivity of 0.47 and specificity of 0.71), and 6.40% for the third trimester (sensitivity of 0.59 and a specificity of 0.63) (Supplementary data 2 (13)).

We also analyzed the relationship between HbA1c and individual adverse outcomes (Fig. 3). No association between HbA1c in the first trimester and pre-eclampsia was observed but higher HbA1c during the first trimester was associated with LGA (OR 1.03; 95% CI 1.01-1.05 per 0.1% increase; *P* < .001), prematurity (OR 1.02; 95% CI 1.01-1.04 per 0.1% increase; *P* = .006), and cesarean section (OR 1.02; 95% CI 1.00-1.03 per 0.1% increase; *P* = .012). In the third trimester, higher HbA1c was associated with LGA (OR 1.07; 95% CI 1.05-1.10 per 0.1% increase; *P* < .001) and prematurity (OR 1.04; 95% CI 1.02-1.07 per 0.1% increase; *P* < .001). The association between HbA1c during the third trimester and cesarean section was borderline significant (OR 1.02; 95% CI 1.00-1.04 per 0.1% increase; *P* = .108). These associations were unaltered after adjustment for year of pregnancy and after adjustment for treatment. No association between the difference in HbA1c between the first and third trimesters and LGA, prematurity, pre-eclampsia, or cesarean section was observed.

Four glycemic control subgroups, defined by HbA1c levels in the first and third trimesters, were created in order to define the period during which HbA1c was a better predictor of maternal–fetal morbidity: group I was optimal balance throughout pregnancy and included 21.6% of the cohort and was considered the reference group; group II had early imbalance but progressive correction during pregnancy and represented 7.6% of the population; group III had a late glycemic imbalance in the third trimester, representing 18.3% of the population; and group IV had glycemic imbalance throughout pregnancy, making up 52.5% of the population. Associations of HbA1c with CC and most individual adverse events, according to these glycemic control levels, are presented in Table 3. Using patients from group I as reference, group II was associated with an increased rate of the CC (OR 2.81; 95% CI 1.01-7.86) and an increased rate of LGA (OR 2.20; 95% CI 1.01-4.78). Patients from group IV were also associated with an increased rate of the CC (OR 2.69; 95% CI 1.58-4.56) and an increased rate of LGA (OR 3.05; 95% CI 1.92-4.87), using patients from group I as reference. Patients from group III were not associated with an increased rate of the CC (OR 1.36; 95% CI 0.73-2.53) nor with an increased rate of LGA (OR 1.62; 95% CI 0.92-2.84). After adjustment for year of the date of pregnancy, patients from group II were still associated with an increased rate of the CC (adjusted OR; 3.54 95% CI 1.18-10.6) or with an increased rate of LGA (adjusted OR 2.41; 95% CI 1.01-5.73). No association between these groups and prematurity or cesarean section was observed. Excluding cesarean sections from the CC did not modify the results (data not shown).

**Table 3.** Association of HbA1c with composite criterion and most individual adverse events according to glycemic control subgroups

	Group I (n = 113)	Group II (n = 40)	Group III (n = 96)	Group IV (n = 274)	*P*
Composite criterion					
No./Total no. (%)	75/109 (68.81)	31/36 (86.11)	69/92 (75)	231/270 (85.56)	
Unadjusted OR (95% CI)	1.00 (ref)	2.81 (1.01-7.86)	1.36 (0.73-2.53)	2.69 (1.58-4.56)	.001
Adjusted^*a*^ OR (95% CI)	1.00 (ref)	3.54 (1.18-10.6)	1.25 (0.63-2.47)	3.20 (1.76-5.82)	<.001
**LGA**					
No./Total no. (%)	40/106 (37.74)	20/35 (57.14)	46/93 (49.46)	174/268 (64.93)	
Unadjusted OR (95% CI)	1.00 (ref)	2.20 (1.01-4.78)	1.62 (0.92-2.84)	3.05 (1.92-4.87)	<.001
Adjusted^*a*^ OR (95% CI)	1.00 (ref)	2.41 (1.01-5.73)	1.46 (0.79-2.69)	3.38 (2.00-5.70)	<.001
Prematurity					
No./Total no. (%)	16/110 (14.55)	7/38 (18.42)	18/95 (18.95)	60/271 (22.14)	
Unadjusted OR (95% CI)	1.00 (ref)	1.33 (0.50-3.52)	1.37 (0.66-2.87)	1.67 (0.91-3.05)	.41
Adjusted^*a*^ OR (95% CI)	1.00 (ref)	1.39 (0.50-3.85)	1.21 (0.56-2.59)	1.95 (1.03-3.70)	.16
Cesarean section					
No./Total no. (%)	44/112 (39.29)	19/38 (50)	42/95 (44.21)	134/274 (48.91)	
Unadjusted OR (95% CI)	1.00 (ref)	1.55 (0.74-3.24)	1.23 (0.70-2.13)	1.48 (0.95-2.31)	.35
Adjusted^*a*^ OR (95% CI)	1.00 (ref)	1.44 (0.67-3.13)	1.27 (0.71-2.25)	1.57 (0.97-2.53)	.32

Values are expressed as the number/total number (%) and as OR (95% CI).

The composite criterion of complications is defined by the presence of at least 1 of the 5 following complications: pre-eclampsia, LGA, SGA, cesarean, prematurity. The different groups are defined as: group I, HbA1c at first trimester <6.5% and <6% at third trimester, considered as reference group; group II, HbA1c at first trimester ≥6.5% and < 6% third trimester; group III, HbA1c at first trimester <6.5% and >6% at third trimester; group IV, HbA1c at first trimester ≥6.5% and ≥6% at third trimester.

Abbreviations: CI, confidence interval; LGA, large for gestational age; SGA, small for gestational age.

^*a*^Adjusted for years of the date of pregnancy

*P* for global comparisons.

## Discussion

The aim of this study was to define if there is an association between HbA1c levels and maternal–fetal complications in T1D. In this large cohort, we found that HbA1c was associated with complications and that early HbA1c was able to predict several adverse outcomes including LGA, SGA, pre-eclampsia, and preterm delivery. Interestingly, results showed that improvement in HbA1c levels between the first and third trimester is not sufficient to have the same rate of LGA as the background population.

Before conception, our large cohort had similar characteristics to other published cohorts in terms of age, BMI, duration of diabetes, and the presence of diabetic vascular complications. Similar to other studies, only one-quarter of our population had HbA1c levels in the target range at conception (14). Indeed, The American Diabetes Association recommends an HbA1c ≤6.5% (48 mmol/mol) before pregnancy or <6% (42 mmol/mol) in the absence of hypoglycemia (7). The small number of women with ideal HbA1c levels at conception may suggest a failure of, or noncompliance with, preconception counseling and programming of pregnancy. Publications have confirmed the impact of good metabolic control prior to pregnancy on maternal–fetal morbidity (15). As expected, conception levels of HbA1c gradually improved over time in our study. In our cohort, 40.8% of women had an HbA1c level ≤7% (53 mmol/mol) in early pregnancy, allowing us to evaluate this point.

Despite a gradual improvement in glycemic balance, as shown by HbA1c, many complications are still reported in pregnancy. In our study, we have demonstrated that the rate of CC was above 80%. There is currently no consensus regarding the CC of choice for assessing maternal–fetal morbidity. We focused on the complications that seemed most important either in terms of frequency or severity. A recent study of 488 pregnant women with T1D reported good outcomes in 44% of pregnancies, but included only uncomplicated deliveries of normal infants, non-LGA after spontaneous labor, and no perinatal complications (16). This study also showed a continuous association between good perinatal outcomes and HbA1c at delivery, suggesting that the lower the HbA1c the better the perinatal outcome. They reported that HbA1c <6.0% (42 mmol/mol) at delivery, which was achieved in 41% of women, was identified by ROC analysis as the best threshold for predicting good outcomes. However, this threshold does not allow accurate identification of infants at low risk of perinatal complications since an adverse perinatal outcome was observed in 40% of women with HbA1c <6.0% (42 mmol/mol) at delivery and, conversely, good perinatal outcomes were noted in 32% of women with HbA1c of >6.0% (42 mmol/mol) at delivery.

Our results suggest that when all complications are combined, the CC was associated with HbA1c in the first trimester and the third trimester, with a greater risk of onset when the HbA1c level was high. We confirm that the tighter the glycemic control, the lower the risk of maternal–fetal complications. Our findings are in agreement with those of Owens et al., who demonstrated in a cohort of 323 diabetic women, including 215 with T1D, an association between HbA1c and maternal and fetal complications, including LGA, preterm delivery, cesarean section, and pre-eclampsia (5). This is consistent with other published data (17-19). However, they reported a cut-off of HbA1c >6.8% (51 mmol/mol) that could predict this comorbidity, which is higher than the value we found, while also finding an equally weak sensitivity/specificity. Despite the large cohort, ROC curves in our study did not show sufficiently sensitivity and specificity to predict complications of pregnancy. It seems likely that the variables included in our CC may explain this result. A CC must be defined which includes complications associated with a higher risk for the mother and the fetus. To develop this criterion, we carried out the same analyses while excluding cesarean section, and this did not modify our results, with an added risk of maternal–fetal complications still found when there was an early glycemic imbalance, despite this being corrected during pregnancy. The impact on maternal–fetal complications of HbA1c levels in the first trimester has been infrequently described in the literature (9, 20), and even less so in women with T1D (21, 22). The data are weak and sometimes contradictory, and it is important to note that the distinction between type 1 and type 2 diabetes is rarely made. A 5-year cohort study based on subjects with type 1 and type 2 diabetes highlighted a cut-off for HbA1c in the third trimester of >6.6% as an independent risk factor for perinatal mortality (23). Similarly, the impact of HbA1c in the third trimester has been little described. However, a recent Qatari study reported different results; similar to our results they highlighted an association between LGA and HbA1c in the third trimester but also a reduction in the risk of LGA which was greater when there was significant change in HbA1c between first and third trimesters. The impact of ethnicity and initial glycemic imbalance (more severe with an Hba1c of 7.9% [63 mmol/mol] vs 6.7% [50 mmol/mol] in our study) in the first trimester should not be ignored (24). Conversely, Abell et al. reported a diminution in the risk of adverse outcomes when HbA1c was higher in early pregnancy (25). These patients received early care which reduced this risk by improving glycemic control. The most important novel finding in our study is that among women with poorer metabolic control at the beginning of pregnancy, the risk of complications persists even if we improve the HbA1c. To follow the American Diabetes Association recommendations, a target of HbA1c <6% should be reached, from the first trimester, limiting the risk of maternal hypoglycemia (7). We conducted the same analyses in our population with these ideal targets; the results were similar (supplementary data 3 (13)). Unfortunately, the retrospective nature of the study did not allow us to collect data on maternal hypoglycemia, especially severe hypoglycemia. However, in our cohort, the variation of HbA1c between the first and third trimesters was not associated with this criterion, which led us to evaluate each element of this CC independently in each period, with LGA, prematurity, and cesarean section being associated with HbA1c levels in the first and also the third trimester. Only pre-eclampsia was not significantly associated with HbA1c. In addition to the low number of cases of pre-eclampsia, our data differ from most published data (26). During this first stage, we noted the absence of an association between its complications and first- to third-trimester variation of HbA1c, suggesting that correction of glycemic balance after the first trimester would have less impact on this morbidity.

Our study has several strengths including the large sample size of T1D subjects and their evaluation by the same multidisciplinary team, their clinical characteristics being in accordance with the literature. This provides statistical power and constitutes the main strong point of the study. In addition, a single laboratory performing HbA1c measurements provides a robust data set. In our clinical practice, we take an HbA1c test every month during pregnancy. So taking HbA1c each month of each quarter allowed us to obtain medians of HbA1c during each trimester, thus limiting the risk of error induced by a single assay, which would be erroneous for different reasons. However, the collection of monocentric data, the particularly long duration, and the retrospective design of this study with some missing data could generate a lack of power in statistical comparisons. Furthermore, some potential limitations should be discussed. Firstly, hemoglobin and mean corpuscular volume levels in our patients were unknown, thus we were unable to exclude the presence of hemoglobinopathy, iron deficiency, or anemia, which can impact the accuracy of HbA1c assessments during pregnancy (27). However, we have a lower rate of hemoglobinopathy in our center. Secondly, with reference to the CONCEPTT study, diabetes management evolved over the years of the study with the increasingly frequent use of an insulin pump, continuous glucose monitoring systems, and the use of new insulin analogs (6). However, even if the management of diabetes changed over the time, such as analogs vs nonanalogs, multiple MDI vs CSII, we have adjusted on the year of pregnancy and the type of treatment. We have demonstrated that our results were not significantly different. Third, we are well aware of the pitfall of not having data on neonatal hypoglycemia. The main reason is related to the difficulties of collecting this criterion because it is a retrospective study. Indeed, between 1997 and 2019, the modalities of research of neonatal hypoglycemia have evolved: research rarely performed initially and systematically now in all newborns of mothers with diabetes.

In conclusion, HbA1c, even if little used in clinical practice, could be a marker for monitoring glycemic balance during pregnancy. Elevated HbA1c is associated with numerous maternal–fetal complications during the first and also during the third trimester. Improving glycemic balance seems to only partially reduce this risk without eliminating it, suggesting the involvement of other associated mechanisms, for example BMI (28), immune mechanisms (29), and glycemic variability (30).

## Data Availability

Some or all datasets generated during and/or analyzed during the current study are not publicly available but are available from the corresponding author on reasonable request.

## Abbreviations

AUC: area under the curve
BW: birthweight
BMI: body mass index
CC: composite criterion
CSII: continuous subcutaneous insulin infusion
CGM: continuous glucose monitoring
HbA1c: glycated hemoglobin
IQR: interquartile range
LGA: large for gestational age
MDI: multiple daily injection
ROC: receiver operating characteristic
SGA: small for gestational age
T1D: type 1 diabetes

## References

[1] Saeedi P , Petersohn I, Salpea P, et al.; IDF Diabetes Atlas Committee. Global and regional diabetes prevalence estimates for 2019 and projections for 2030 and 2045: results from the International Diabetes Federation Diabetes Atlas, 9th edition. Diabetes Res Clin Pract. 2019;157:107843. 31518657 10.1016/j.diabres.2019.107843

[2] Schaefer-Graf U , Napoli A, Nolan CJ; Diabetic Pregnancy Study Group. Diabetes in pregnancy: a new decade of challenges ahead. Diabetologia. 2018;61(5):1012-1021. 29356835 10.1007/s00125-018-4545-y PMC6448995

[3] Durackova L , Kristufkova A, Korbel M. Pregnancy and neonatal outcomes in women with type 1 diabetes mellitus. Bratisl Lek Listy. 2017;118(1):56-60. 28127984 10.4149/BLL_2017_011

[4] Diabetes care and research in Europe: the Saint Vincent declaration. Diabet Med. 1990;7(4):360. 2140091

[5] Owens LA , Sedar J, Carmody L, Dunne F. Comparing type 1 and type 2 diabetes in pregnancy- similar conditions or is a separate approach required? BMC Pregnancy Childbirth. 2015;15:69. 25885892 10.1186/s12884-015-0499-y PMC4390076

[6] Feig DS , Donovan LE, Corcoy R, et al.; CONCEPTT Collaborative Group. Continuous glucose monitoring in pregnant women with type 1 diabetes (CONCEPTT): a multicentre international randomised controlled trial. Lancet. 2017;390(10110):2347-2359. 28923465 10.1016/S0140-6736(17)32400-5 PMC5713979

[7] American Diabetes Association. 14. Management of diabetes in pregnancy: standards of medical care in diabetes-2021. Diabetes Care. 2021;44(Suppl 1):S200-S210. 33298425 10.2337/dc21-S014

[8] Rafat D , Ahmad J. HbA1c in pregnancy. Diabetes Metab Syndr. 2012;6(1):59-64. 23014257 10.1016/j.dsx.2012.05.010

[9] Mañé L , Flores-Le Roux JA, Gómez N, et al. Association of first-trimester HbA1c levels with adverse pregnancy outcomes in different ethnic groups. Diabetes Res Clin Pract. 2019;150:202-210. 30880095 10.1016/j.diabres.2019.03.017

[10] O’Connor C , O’Shea PM, Owens LA, et al. Trimester-specific reference intervals for haemoglobin A1c (HbA1c) in pregnancy. Clin Chem Lab Med. 2011;50(5):905-909. 22117781 10.1515/CCLM.2011.397

[11] Bismuth E , Bouche C, Caliman C, et al.; French-Speaking Diabetes Society. Management of pregnancy in women with type 1 diabetes mellitus: guidelines of the French-Speaking Diabetes Society (Société francophone du diabète [SFD]). Diabetes Metab. 2012;38(3):205-216. 22521040 10.1016/j.diabet.2012.02.010

[12] Mamelle N , Munoz F, Grandjean H. Fetal growth from the AUDIPOG study. I. Establishment of reference curves. J Gynecol Obstet Biol Reprod (Paris). 1996;25(1):61-70. 8901304

[13] Lemaitre M , Vambergue A. Association between HbA1c levels on adverse pregnancy outcomes during pregnancy in type 1 diabetic patients. Dryad. Dataset. 2021. 10.5061/dryad.2fqz612q2 PMC8852207 34694409

[14] Bernasko J . Contemporary management of type 1 diabetes mellitus in pregnancy. Obstet Gynecol Surv. 2004;59(8):628-636. 15277897 10.1097/00006254-200408000-00024

[15] Kekäläinen P , Juuti M, Walle T, Laatikainen T. Pregnancy planning in type 1 diabetic women improves glycemic control and pregnancy outcomes. J Matern Fetal Neonatal Med. 2016;29(14):2252-2258. 26364952 10.3109/14767058.2015.1081888

[16] Lepercq J , Le Ray C, Godefroy C, Pelage L, Dubois-Laforgue D, Timsit J. Determinants of a good perinatal outcome in 588 pregnancies in women with type 1 diabetes. Diabetes Metab. 2019;45(2):191-196. 29776801 10.1016/j.diabet.2018.04.007

[17] McGrath RT , Glastras SJ, Hocking SL, Fulcher GR. Large-for-gestational-age neonates in type 1 diabetes and pregnancy: contribution of factors beyond hyperglycemia. Diabetes Care. 2018;41(8):1821-1828. 30030258 10.2337/dc18-0551

[18] Reece EA , Homko CJ. Diabetes-related complications of pregnancy. J Natl Med Assoc. 1993;85(7):537-545. 8350376 PMC2568145

[19] Bashir M , Naem E, Taha F, Konje JC, Abou-Samra AB. Outcomes of type 1 diabetes mellitus in pregnancy; effect of excessive gestational weight gain and hyperglycaemia on fetal growth. Diabetes Metab Syndr. 2019;13(1):84-88. 30641818 10.1016/j.dsx.2018.08.030

[20] Versantvoort AR , van Roosmalen J, Radder JK. Course of HbA1c in non-diabetic pregnancy related to birth weight. Neth J Med. 2013;71(1):22-25. 23412819

[21] Nielsen GL , Møller M, Sørensen HT. HbA1c in early diabetic pregnancy and pregnancy outcomes: a Danish population-based cohort study of 573 pregnancies in women with type 1 diabetes. Diabetes Care. 2006;29(12):2612-2616. 17130193 10.2337/dc06-0914

[22] Starikov RS , Inman K, Chien EK, et al. Can hemoglobin A1c in early pregnancy predict adverse pregnancy outcomes in diabetic patients? J Diabetes Complications. 2014;28(2):203-207. 24268941 10.1016/j.jdiacomp.2013.10.004

[23] Murphy HR , Howgate C, O’Keefe J, et al.; National Pregnancy in Diabetes (NPID) advisory group. Characteristics and outcomes of pregnant women with type 1 or type 2 diabetes: a 5-year national population-based cohort study. Lancet Diabetes Endocrinol. 2021;9(3):153-164. 33516295 10.1016/S2213-8587(20)30406-X

[24] Bashir M , Naem E, Taha F, Konje JC, Abou-Samra AB. Outcomes of type 1 diabetes mellitus in pregnancy; effect of excessive gestational weight gain and hyperglycaemia on fetal growth. Diabetes Metab Syndr. 2019;13(1):84-88. 30641818 10.1016/j.dsx.2018.08.030

[25] Abell SK , Boyle JA, de Courten B, et al. Contemporary type 1 diabetes pregnancy outcomes: impact of obesity and glycaemic control. Med J Aust. 2016;205(4):162-167. 27510344 10.5694/mja16.00443

[26] Vestgaard M , Sommer MC, Ringholm L, Damm P, Mathiesen ER. Prediction of preeclampsia in type 1 diabetes in early pregnancy by clinical predictors: a systematic review. J Matern Fetal Neonatal Med. 2018;31(14):1933-1939. 28574296 10.1080/14767058.2017.1331429

[27] Renz PB , Hernandez MK, Camargo JL. Effect of iron supplementation on HbA1c levels in pregnant women with and without anaemia. Clin Chim Acta. 2018;478:57-61. 29274326 10.1016/j.cca.2017.12.028

[28] Hidayat K , Zou SY, Shi BM. The influence of maternal body mass index, maternal diabetes mellitus, and maternal smoking during pregnancy on the risk of childhood-onset type 1 diabetes mellitus in the offspring: Systematic review and meta-analysis of observational studies. Obes Rev. 2019;20(8):1106-1120. 31090253 10.1111/obr.12858

[29] Groen B , Links TP, van den Berg PP, de Vos P, Faas MM. The role of autoimmunity in women with type 1 diabetes and adverse pregnancy outcome: a missing link. Immunobiology. 2019;224(2):334-338. 30819511 10.1016/j.imbio.2019.02.003

[30] Mulla BM , Noor N, James-Todd T, et al. Continuous glucose monitoring, glycemic variability, and excessive fetal growth in pregnancies complicated by type 1 diabetes. Diabetes Technol Ther. 2018;20(6):413-419. 29901410 10.1089/dia.2017.0443 PMC6014051

